# Zinc Ameliorates the Osteogenic Effects of High Glucose in Vascular Smooth Muscle Cells

**DOI:** 10.3390/cells10113083

**Published:** 2021-11-09

**Authors:** Laura A. Henze, Misael Estepa, Burkert Pieske, Florian Lang, Kai-Uwe Eckardt, Ioana Alesutan, Jakob Voelkl

**Affiliations:** 1 Department of Internal Medicine and Cardiology, Charité—Universitätsmedizin Berlin, Campus Virchow-Klinikum, 13353 Berlin, Germany; laura.henze@charite.de (L.A.H.); misael.estepa@gmail.com (M.E.); burkert.pieske@charite.de (B.P.); 2 Department of Vegetative and Clinical Physiology, Eberhard Karls University Tübingen, 72076 Tübingen, Germany; florian.lang@uni-tuebingen.de; 3 Department of Nephrology and Medical Intensive Care, Charité—Universitätsmedizin Berlin, 13353 Berlin, Germany; kai-uwe.eckardt@charite.de (K.-U.E.); jakob.voelkl@jku.at (J.V.); 4 Institute for Physiology and Pathophysiology, Johannes Kepler University Linz, 4040 Linz, Austria; 5 DZHK (German Centre for Cardiovascular Research), Partner Site Berlin, 13347 Berlin, Germany

**Keywords:** diabetes mellitus, GPR39, high glucose, osteogenic transition, vascular calcification, vascular smooth muscle cells, zinc, NF-kB

## Abstract

In diabetic patients, medial vascular calcification is common and associated with increased cardiovascular mortality. Excessive glucose concentrations can activate the nuclear factor kappa-light-chain-enhancer of activated B-cells (NF-kB) and trigger pro-calcific effects in vascular smooth muscle cells (VSMCs), which may actively augment vascular calcification. Zinc is able to mitigate phosphate-induced VSMC calcification. Reduced serum zinc levels have been reported in diabetes mellitus. Therefore, in this study the effects of zinc supplementation were investigated in primary human aortic VSMCs exposed to excessive glucose concentrations. Zinc treatment was found to abrogate the stimulating effects of high glucose on VSMC calcification. Furthermore, zinc was found to blunt the increased expression of osteogenic and chondrogenic markers in high glucose-treated VSMCs. High glucose exposure was shown to activate NF-kB in VSMCs, an effect that was blunted by additional zinc treatment. Zinc was further found to increase the expression of TNFα-induced protein 3 (TNFAIP3) in high glucose-treated VSMCs. The silencing of TNFAIP3 was shown to abolish the protective effects of zinc on high glucose-induced NF-kB-dependent transcriptional activation, osteogenic marker expression, and the calcification of VSMCs. Silencing of the zinc-sensing receptor G protein-coupled receptor 39 (GPR39) was shown to abolish zinc-induced *TNFAIP3* expression and the effects of zinc on high glucose-induced osteogenic marker expression. These observations indicate that zinc may be a protective factor during vascular calcification in hyperglycemic conditions.

## 1. Introduction

Vascular calcification is closely associated with a high risk of cardiovascular events and mortality [1,2]. In patients with diabetes mellitus, vascular calcification is accelerated [3,4,5,6] and may contribute to the development of cardiovascular diseases [3], which in turn are responsible in large part for the high mortality of these patients [3,7]. Nonetheless, no broadly applicable treatment against the development of vascular calcification is currently available [1,8].

Vascular calcification is an active process [9], involving a transition of vascular smooth muscle cells (VSMCs) into cells with some osteoblast- and chondroblast-like characteristics [9,10]. These VSMCs express increased levels of osteogenic and chondrogenic transcription factors, such as core-binding factor alpha-1 (CBFA1/RUNX2) or SRY-box transcription factor 9 (SOX9), as well as enzymes such as tissue-nonspecific alkaline phosphatase (ALPL) [9,10,11]. These cells contribute to the creation of a pro-calcific environment in the media of the vasculature and actively promote tissue mineralization [9]. Hyperglycemia has been suggested to be a key pathological factor that triggers the expression of osteogenic markers in VSMCs [12,13,14] and augments vascular calcification [12,13,14,15].

The reprogramming of VSMCs is controlled by complex intracellular signaling pathways [9,16,17]. A key mediator of the pro-calcific effects of high glucose in VSMCs is the transcription factor NF-kB (nuclear factor kappa-light-chain-enhancer of activated B-cells) [14,18]. High levels of glucose activate NF-kB in VSMCs [14,19,20]. Interference with NF-kB activation can suppress high glucose-induced osteogenic signaling in VSMCs [14]. Zinc may act as an inhibitor of NF-kB activity in VSMCs [21]. Zinc supplementation can ameliorate VSMC calcification during phosphate exposure [21,22] and valvular interstitial cell calcification [23].

Zinc has been associated with beta cell function and insulin resistance [24] and reduced serum zinc levels are observed in patients with diabetes mellitus [25,26,27,28,29], most likely due to hyperzincuria and intestinal malabsorption [25,26,30]. Zinc deficiency is a risk factor for diabetes [31] and its cardiovascular complications [32]. Moreover, in patients with diabetes mellitus, serum zinc levels inversely associate with serum calcification propensity, indicating an increased risk of calcification [33]. However, the direct impact of zinc on vascular calcification during diabetic conditions is still unclear. Therefore, the current study explored the effects of zinc supplementation on osteogenic/chondrogenic marker expression and the calcification of VSMCs promoted by elevated glucose levels in vitro.

## 2. Materials and Methods

### 2.1. Primary Human Aortic Smooth Muscle Cells (HAoSMCs)

HAoSMCs (Fisher Scientific, Vienna, Austria and Sigma Aldrich, Vienna, Austria) were cultured as previously described [34,35] and used in experiments up to passage 11. HAoSMCs were treated with 50 mM of glucose (Sigma Aldrich, Vienna, Austria) [14], 15 µM of ZnSO_4_ (Sigma Aldrich, Vienna, Austria), and 15 µM of ZnCl_2_ (Sigma Aldrich, Vienna, Austria) [21]. For calcification analysis, HAoSMCs were treated for 11 days with calcification medium supplemented with 10 mM of β-glycerophosphate and 1.5 mM of CaCl_2_ (Sigma-Aldrich, Vienna, Austria) [36,37,38]. Fresh medium with agents was added every two to three days. Where indicated, HAoSMCs were transfected with 10 nM of TNFAIP3 (ID: s14260), GPR39 (ID: s6074) or negative control (ID: 4390843) siRNA using siPORT amine transfection reagent (all from Fisher Scientific, Vienna, Austria) [21].

### 2.2. RT-PCR

Total RNA was isolated from HAoSMCs using trizol reagent [34,39] and cDNA was synthesized with oligo(dT)_12–18_ primers and SuperScript III Reverse Transcriptase (all from Fisher Scientific, Vienna, Austria). RT-PCR was performed using iQ Sybr Green Supermix (Bio-Rad Laboratories, Vienna, Austria) and the following human primers (Fisher Scientific, Vienna, Austria) [14,21,40]:

*CBFA1* fw: GCCTTCCACTCTCAGTAAGAAGA;
*CBFA1* rev: GCCTGGGGTCTGAAAAAGGG;
*SOX9* fw: AGCGAACGCACATCAAGAC;
*SOX9* rev: CTGTAGGCGATCTGTTGGGG;
*ALPL* fw: GGGACTGGTACTCAGACAACG;
*ALPL* rev: GTAGGCGATGTCCTTACAGCC;
*TNFAIP3* fw: TCAACTGGTGTCGAGAAGTCC;
*TNFAIP3* rev: CAAGTCTGTGTCCTGAACGC;
*GPR39* fw: TCTTCGTGATGGGCCTTCTG;
*GPR39* rev: ACCTCCTTCTGCAAGTATCCTTT;
*GAPDH* fw: GAGTCAACGGATTTGGTCGT;
*GAPDH* rev: GACAAGCTTCCCGTTCTCAG.

A melt curve analysis was performed to confirm the specificity of the PCR products. The relative mRNA expression was determined using the 2^−ΔΔCt^ method with GAPDH as the housekeeping gene, normalized to the control group.

### 2.3. Western Blotting

Proteins were isolated from HAoSMCs using ice-cold Pierce IP lysis buffer containing complete protease and a phosphatase inhibitor cocktail (all from Fisher Scientific, Vienna, Austria). Protein concentration was measured using the Bradford assay (Bio-Rad Laboratories, Vienna, Austria) [37,38]. Equal amounts of proteins incubated in Roti-Load1 Buffer (Carl Roth, Karlsruhe, Germany) for 10 min at 100 °C were separated on SDS-PAGE gels and transferred to PVDF membranes. Membranes were incubated with primary rabbit anti-RUNX2 (1:1000, Cell Signaling, Frankfurt am Main, Germany, #8486) or rabbit anti-GAPDH (1:1000, Cell Signaling, Frankfurt am Main, Germany, #2118) antibodies overnight at 4 °C and then with secondary anti-rabbit HRP-conjugated antibody (1:1000, Cell Signaling, Frankfurt am Main, Germany) for 1 h at room temperature, before being stripped in stripping buffer (Fisher Scientific, Vienna, Austria) at room temperature. Bands were detected with the ECL detection reagent (Fisher Scientific, Vienna, Austria) and quantified using the ImageJ software (NIH, Rockville, MD, USA, 1.52n). Data are shown as the ratio of total protein to GAPDH and were normalized to the control group [14,21].

### 2.4. NF-kB Activity

NF-kB-dependent transcriptional activity in HAoSMCs was determined using equal amounts of nuclear proteins, isolated with the NE-PER nuclear and cytoplasmic extraction kit (Fisher Scientific, Vienna, Austria), and quantified by the Bradford assay (Bio-Rad Laboratories, Vienna, Austria) [41] and the NF-kB p65 transcription factor colorimetric assay kit (Abcam, Cambridge, UK) [14]. Data are shown normalized to the control group.

### 2.5. OPN Levels

OPN levels in conditioned medium from HAoSMCs were determined by using the human OPN DuoSet ELISA kit (R&D Systems, Abingdon, UK, #DY1433) and DuoSet Ancillary Reagent kit 2 (R&D Systems, Abingdon, UK). HAoSMCs were lysed with ice-cold Pierce IP lysis buffer containing a complete protease and phosphatase inhibitors cocktail (all from Fisher Scientific, Vienna, Austria). Data are shown normalized to total protein concentration measured by the Bradford assay (Bio-Rad Laboratories, Vienna, Austria) and to the control group.

### 2.6. ALP Activity

ALP enzyme activity was determined in cell lysates using the ALP colorimetric assay kit (Abcam, Cambridge, UK). Data are shown normalized to the total protein concentration measured by the Bradford assay (Bio-Rad Laboratories, Vienna, Austria) and to the control group [14].

### 2.7. Calcification Analysis

HAoSMCs were incubated overnight at 37 °C with OsteoSense 680EX (1:250, Perkin Elmer, Traiskirchen, Austria) [42]. Images were acquired with the ChemiDoc MP imaging system (Bio-Rad Laboratories, Vienna, Austria) with excitation/emission (bandpass) wavelengths of 680/715(30) nm [43]. HAoSMCs were decalcified in 0.6 M HCl at 4 °C overnight and the calcium content was quantified with the QuantiChrom Calcium assay kit (BioAssay Systems, Hayward, CA, USA) [14,21,37]. Proteins were isolated using 0.1 M NaOH/0.1% SDS buffer and quantified by the Bradford assay (Bio-Rad Laboratories, Vienna, Austria). Data are shown normalized to total protein concentration.

### 2.8. Statistics

Data are presented as scatter dot plots and arithmetic means ± SEM and *n* indicate the number of independent experiments performed. The Shapiro–Wilk test was used for normality analysis. Prior to statistical testing, non-normal datasets were transformed (log, sqrt or reciprocal) to provide normality. Statistical testing was assessed using one-way ANOVA with Tukey’s post hoc test for homoscedastic data or the Games–Howell post hoc test for heteroscedastic data and the Steel–Dwass method for non-normal data [21,37,44]. A *p* value of <0.05 was considered statistically significant.

## 3. Results

**Figure 1:**
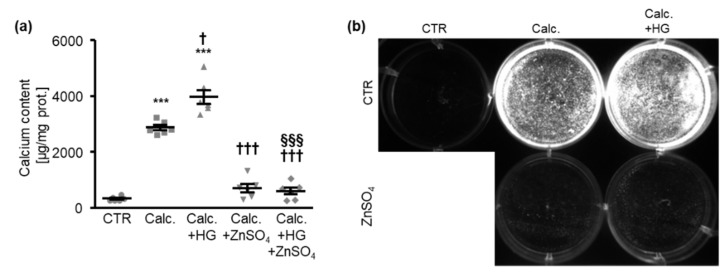
Zinc sulfate inhibits the calcification of HAoSMCs promoted by high levels of glucose. (**a**). Calcium content (*n* = 6, µg/mg protein) in HAoSMCs treated for 11 days with control (CTR) or calcification medium (Calc.) without and with high glucose levels (HG) in the absence and presence of ZnSO_4_. *** (*p* < 0.001) significant difference versus control group; † (*p* < 0.05), ††† (*p* < 0.001) significant difference versus Calc.-treated group; §§§ (*p* < 0.001) significant difference between Calc.+HG- and Calc.+HG+ZnSO_4_-treated groups. (**b**). Calcification detected by fluorescence imaging in HAoSMCs treated for 11 days with control (CTR) or calcification medium (Calc.) without and with high glucose levels (HG) in the absence and presence of ZnSO_4_. Calcified areas: white pseudocolor.

A first series of experiments was used to investigate the effects of zinc sulfate (ZnSO_4_) on the calcification of HAoSMCs during excessive glucose conditions. As shown in Figure 1a, treatment with high level of glucose significantly augmented the calcification of HAoSMCs, which was promoted by exposure to pro-calcific medium containing β-glycerophosphate and calcium as mineralization substrates. Additional treatment with ZnSO_4_ significantly suppressed the calcification of HAoSMCs triggered by calcification medium alone and by calcification medium together with high levels of glucose. In accordance, the fluorescence imaging of the calcification revealed that high-glucose conditions aggravated the calcification of HAoSMCs induced by calcification medium, while ZnSO_4_ supplementation had strong inhibitory effects during these pro-calcific conditions (Figure 1b).

**Figure 2:**
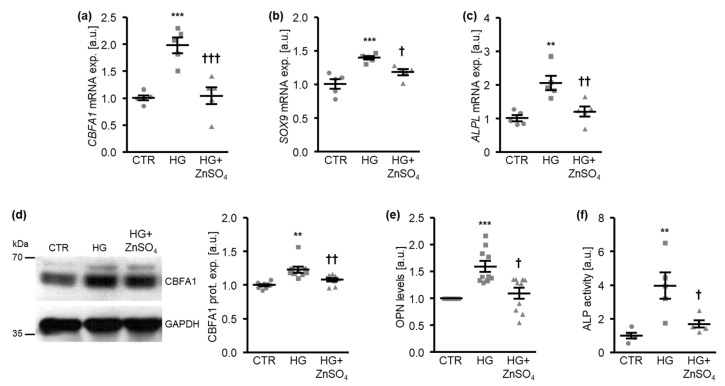
Zinc sulfate suppresses osteo-/chondrogenic marker expression promoted by high glucose levels in HAoSMCs. (**a**–**c**). Relative mRNA expression of *CBFA1* (**a**), *SOX9* (**b**), and *ALPL* (**c**) (*n* = 5; arbitrary units, a.u.) in HAoSMCs treated for 24 h with control (CTR) or high glucose levels (HG) in the absence and presence of ZnSO_4_. (**d**). Representative Western blots and normalized CBFA1 protein expression (*n* = 9; a.u.) in HAoSMCs treated for 24 h with control (CTR) or high glucose levels (HG) in the absence and presence of ZnSO_4_. (**e**). Normalized OPN levels (*n* = 10; a.u.) in conditioned medium from HAoSMCs treated for 7 days with control (CTR) or high glucose levels (HG) in the absence and presence of ZnSO_4_. (**f**). Normalized ALP activity (*n* = 5; a.u.) in HAoSMCs treated for 7 days with control (CTR) or high glucose levels (HG) in the absence and presence of ZnSO_4_. ** (*p* < 0.01), *** (*p* < 0.001) significant difference versus control group; † (*p* < 0.05), †† (*p* < 0.01), ††† (*p* < 0.001) significant difference versus the HG-treated group.

These effects were paralleled by the inhibition of osteo-/chondrogenic signaling in HAoSMCs, as indicated by osteo-/chondrogenic marker expression and activity. High levels of glucose induced *CBFA1*, *SOX9*, and *ALPL* mRNA expression in HAoSMCs, effects that were significantly suppressed by ZnSO_4_ (Figure 2a–c). Moreover, exposure to high levels of glucose significantly increased the CBFA1 protein abundance (Figure 2d), the osteopontin (OPN) levels released in cell culture medium (Figure 2e), as well as the ALP activity (Figure 2f) in HAoSMCs, all effects that were again significantly reduced by ZnSO_4_ supplementation.

**Figure 3:**
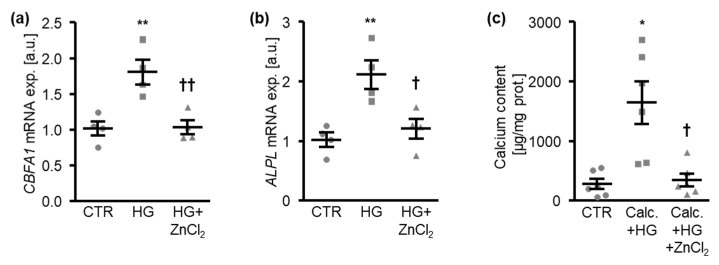
Zinc chloride inhibits osteogenic marker expression and the calcification of HAoSMCs induced by high levels of glucose. (**a**,**b**). Relative mRNA expression of *CBFA1* (**a**) and *ALPL* (**b**) (*n* = 4; arbitrary units, a.u.) in HAoSMCs treated for 24 h with control (CTR) or high glucose levels (HG) in the absence and presence of ZnCl_2_. (**c**). Calcium content (*n* = 6, µg/mg protein) in HAoSMCs treated for 11 days with control (CTR) or calcification medium (Calc.) and high glucose levels (HG) in the absence and presence of ZnCl_2_. * (*p* < 0.05), ** (*p* < 0.01) significant difference versus control group; † (*p* < 0.05), †† (*p* < 0.01) significant difference versus the HG-/Calc.+HG-treated group.

ZnCl_2_ treatment had similar inhibitory effects on the mRNA expression of the osteogenic markers *CBFA1* and *ALPL* (Figure 3a,b) and on the calcification of HAoSMCs (Figure 3c), further confirming the anti-calcific properties of zinc during hyperglycemic conditions. Thus, zinc supplementation inhibited osteo-/chondrogenic reprogramming and the aggravation of calcification of HAoSMCs under high-glucose conditions.

**Figure 4:**
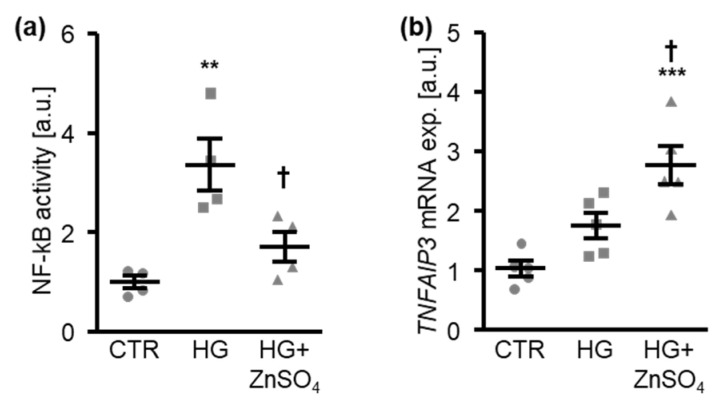
Zinc sulfate suppresses NF-kB activation induced by high levels of glucose in HAoSMCs. (**a**). Normalized NF-kB-dependent transcriptional activity (*n* = 4; arbitrary units, a.u.) in HAoSMCs treated for 30 min with control (CTR) or high glucose levels (HG) in the absence and presence of ZnSO_4_. (**b**). Relative mRNA expression of *TNFAIP3* (*n* = 5; a.u.) in HAoSMCs treated for 24 h with control (CTR) or high glucose levels (HG) in the absence and presence of ZnSO_4_. ** (*p* < 0.01), *** (*p* < 0.001) significant difference versus control group; † (*p* < 0.05) significant difference versus HG-treated group.

To elucidate the underlying mechanisms, the potential interference with NF-kB signaling was analyzed. In HAoSMCs, high levels of glucose significantly increased the NF-kB-dependent transcriptional activity, as indicated by the DNA-binding activity of NF-kB p65 in nuclear extracts, effects that were significantly suppressed in the presence of ZnSO_4_ (Figure 4a). Furthermore, the mRNA expression of TNFα-induced protein 3 (*TNFAIP3*), a suppressor of the NF-kB pathway, was increased by the high glucose treatment and was further up-regulated by the addition of ZnSO_4_ (Figure 4b). Thus, zinc interfered with NF-kB pathway activation in HAoSMCs under high-glucose conditions.

**Figure 5:**
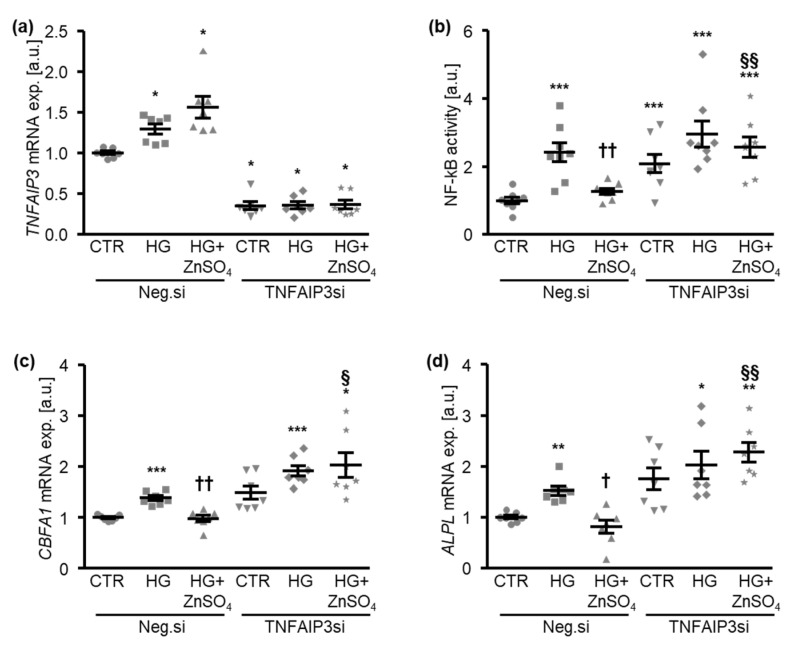
The silencing of TNFAIP3 blocks the effects of zinc sulfate on high glucose-induced NF-kB activation and osteogenic marker expression in HAoSMCs. (**a**). Relative mRNA expression of *TNFAIP3* (*n* = 7; arbitrary units, a.u.) in HAoSMCs transfected with negative control (Neg.si) or TNFAIP3 (TNFAIP3si) siRNA and treated for 24 h with control (CTR) or high glucose levels (HG) in the absence and presence of ZnSO_4_. * (*p* < 0.05) significant difference versus Neg.si-transfected CTR-treated group. (**b**). Normalized NF-kB-dependent transcriptional activity (*n* = 8; a.u.) in HAoSMCs transfected with negative control (Neg.si) or TNFAIP3 (TNFAIP3si) siRNA and treated for 30 min with control (CTR) or high glucose levels (HG) in the absence and presence of ZnSO_4_. (**c**,**d**). Relative mRNA expression of *CBFA1* (**c**) and *ALPL* (**d**) (*n* = 7; a.u.) in HAoSMCs transfected with negative control (Neg.si) or TNFAIP3 (TNFAIP3si) siRNA and treated for 24 h with control (CTR) or high glucose levels (HG) in the absence and presence of ZnSO_4_. * (*p* < 0.05), ** (*p* < 0.01), *** (*p* < 0.001) significant difference versus the Neg.si-transfected CTR-treated group; † (*p* < 0.05), †† (*p* < 0.01) significant difference between the Neg.si-transfected HG- and HG+ZnSO_4_-treated groups; § (*p* < 0.05), §§ (*p* < 0.01) significant difference between the Neg.si- and TNFAIP3si-transfected HG+ZnSO_4_-treated groups.

**Figure 6:**
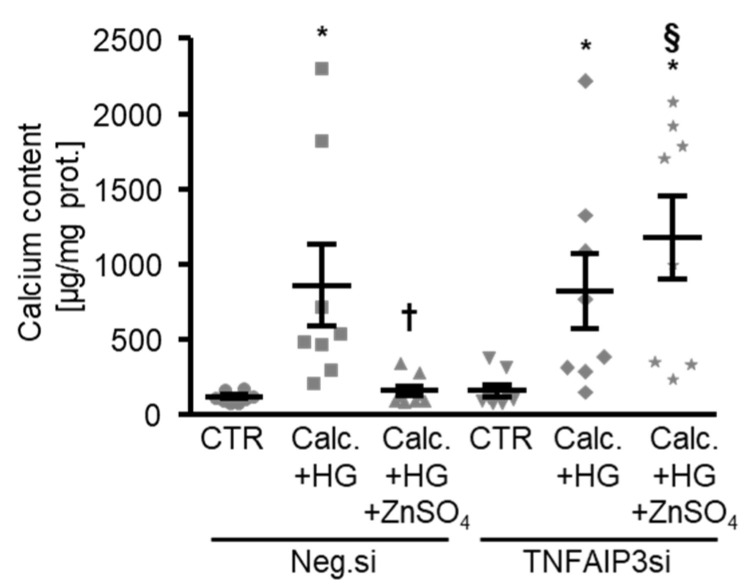
The silencing of TNFAIP3 interferes with the protective effects of zinc sulfate on the calcification of HAoSMCs under high-glucose conditions. Calcium content (*n* = 8, µg/mg protein) in HAoSMCs transfected with negative control (Neg.si) or TNFAIP3 (TNFAIP3si) siRNA and treated for 11 days with control (CTR) or calcification medium (Calc.) and high glucose levels (HG) in the absence and presence of ZnSO_4_. * (*p* < 0.05) significant difference versus the Neg.si-transfected CTR-treated group; † (*p* < 0.05) significant difference between the Neg.si-transfected Calc.+HG- and Calc.+HG+ZnSO_4_-treated groups; § (*p* < 0.05) significant difference between the Neg.si- and TNFAIP3si-transfected Calc.+HG+ZnSO_4_-treated groups.

The next experiments investigated the involvement of TNFAIP3 in the protective effects of zinc during high glucose-induced osteogenic signaling and the calcification of HAoSMCs. To this end, the endogenous expression of TNFAIP3 was suppressed by silencing using small interfering RNA (siRNA) (Figure 5a). The silencing of TNFAIP3 alone was sufficient to significantly increase the NF-kB-dependent transcriptional activity in HAoSMCs (Figure 5b). ZnSO_4_ treatment significantly suppressed the high glucose-induced NF-kB-dependent transcriptional activity in negative control siRNA-transfected HAoSMCs, but not in TNFAIP3-silenced HAoSMCs (Figure 5b). As illustrated in Figure 5c,d, TNFAIP3 knockdown tended to increase the *CBFA1* and *ALPL* mRNA expression in HAoSMCs during control conditions. The difference, however, did not reach statistical significance (*p* = 0.064 and *p* = 0.066, respectively). Moreover, the silencing of TNFAIP3 significantly blunted the inhibitory effects of ZnSO_4_ on the high glucose-induced mRNA expression of osteogenic markers in HAoSMCs (Figure 5c,d). Accordingly, the silencing of TNFAIP3 abrogated the protective effects of ZnSO_4_ supplementation on the calcification of HAoSMCs under high-glucose conditions (Figure 6).

**Figure 7:**
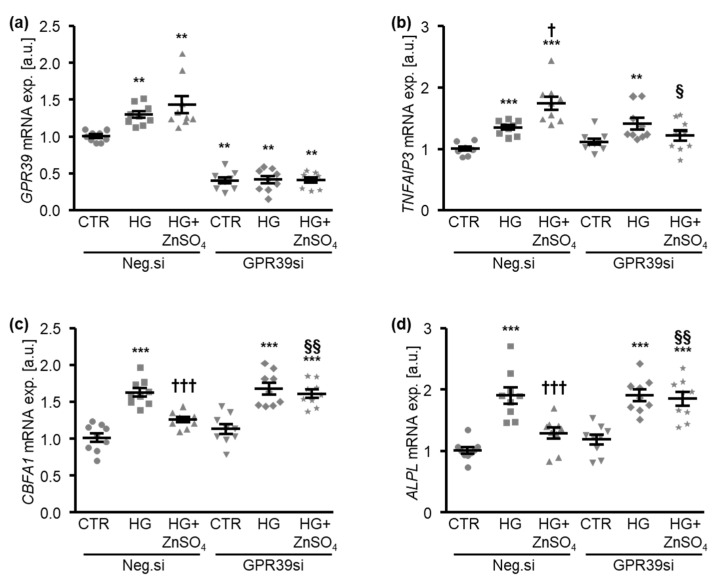
The silencing of GPR39 blunts the protective effects of zinc sulfate on high glucose-induced osteogenic marker expression in HAoSMCs. (**a**). Relative mRNA expression of *GPR39* (*n* = 9; arbitrary units, a.u.) in HAoSMCs transfected with negative control (Neg.si) or GPR39 (GPR39si) siRNA and treated for 24 h with control (CTR) or high glucose levels (HG) in the absence and presence of ZnSO_4_. ** (*p* < 0.01) significant difference versus the Neg.si-transfected CTR-treated group. (**b**–**d**). Relative mRNA expression of *TNFAIP3* (**b**), *CBFA1* (**c**), and *ALPL* (**d**) (*n* = 9; a.u.) in HAoSMCs transfected with negative control (Neg.si) or GPR39 (GPR39si) siRNA and treated for 24 h with control (CTR) or high glucose levels (HG) in the absence and presence of ZnSO_4_. ** (*p* < 0.01), *** (*p* < 0.001) significant difference versus the Neg.si-transfected CTR-treated group; † (*p* < 0.05), ††† (*p* < 0.001) significant difference between the Neg.si-transfected HG- and HG+ZnSO_4_-treated groups; § (*p* < 0.05), §§ (*p* < 0.01) significant difference between the Neg.si- and GPR39si-transfected HG+ZnSO_4_-treated groups.

Additional experiments explored the potential role of the zinc-sensing receptor G protein-coupled receptor 39 (GPR39) in mediating the anti-calcific effects of zinc on HAoSMCs by the inhibition of GPR39 endogenous expression using siRNA (Figure 7a). Surprisingly, high levels of glucose up-regulated the mRNA expression of *GPR39* in negative control siRNA-transfected HAoSMCs, effects that were not significantly modified by additional treatment with ZnSO_4_ (Figure 7a). The silencing of GPR39 did not significantly affect the *TNFAIP3*, *CBFA1*, and *ALPL* mRNA expression during control conditions but blunted the up-regulation of *TNFAIP3* and down-regulation of *CBFA1* and *ALPL* mRNA expression in HAoSMCs induced by ZnSO_4_ in the presence of high levels of glucose (Figure 7b–d). Taken together, the protective effects of zinc on the high glucose-induced osteogenic expression profile of HAoSMCs were mediated, at least in part, by the GPR39-dependent up-regulation of *TNFAIP3*.

## 4. Discussion

The present study disclosed the inhibitory effects of zinc supplementation on vascular calcification during excessive glucose concentrations in vitro. Zinc has emerged as a putative protective factor in cardiovascular calcification, and low dietary zinc intake has been linked to calcification in the general population [21,23,45]. In patients with chronic kidney disease, zinc deficiency is associated with the development of cardiovascular disease [46]. The current study extends these findings by showing that zinc deficiency may contribute to vascular calcification during hyperglycemic conditions.

In diabetic patients, at least in skeletal muscle biopsies, an increased activity of NF-kB can be observed after LPS challenge [47]. NF-kB activation has been considered to play a critical role in the detrimental effects of diabetic conditions on VSMCs [14,19,20]. The pro-calcific changes in VSMCs after excessive glucose exposure [15] are associated with the activation of the NF-kB pathway [14]. The current observations indicate that zinc supplementation interferes with the augmenting effects of excessive glucose concentrations on NF-kB activation, osteogenic marker expression, and calcification in VSMCs. The inhibitory effects on NF-kB activation have been discussed as a decisive mechanism underlying the anti-inflammatory effects of zinc [48]. The anti-calcific effects of zinc are at least partly mediated by GPR39, a G protein-coupled receptor that is activated by extracellular zinc and widely associated with anti-inflammatory effects [49]. The activation of GPR39 has been associated with reduced monocyte-to-endothelial adhesion [50] and reduced cardiovascular calcification [21,23]. GPR39 agonism further ameliorates the extracellular matrix degradation in chondrocytes induced by advanced glycation end-products [51]. The anti-inflammatory effects of GPR39 activation can be observed in fibroblast-like synoviocytes after TNFα treatment [52]. Interestingly, GPR39 deficiency is associated with impaired bone composition in mice [53] and zinc deficiency reduces bone mineral density in rats [54], which may indicate the role of zinc in the bone–vascular axis [55].

TNFAIP3, also known as A20, acts as an anti-inflammatory signalling protein and may interfere with NF-kB activation by modulating the ubiquitination or inhibition of the IkB kinase complex [48,56]. Impaired TNFAIP3 expression increases inflammation and transplant arteriosclerosis in mice [57]. TNFAIP3, in turn, is up-regulated by stimulators of NF-kB as part of a negative feedback loop [58], which could explain the increased TNFAIP3 expression in VSMCs exposed to high-glucose conditions. Zinc up-regulates the expression of TNFAIP3 [48], and the inhibitory effects of zinc on high glucose-induced NF-kB activity are dependent on TNFAIP3. A role for TNFAIP3 in zinc-mediated NF-kB inhibition has also been shown in endothelial [59] and microglial [60] cells. TNFAIP3 expression is reduced in peripheral blood mononuclear cells (PBMCs) from patients with type 2 diabetes [61]. Thus, the up-regulation of TNFAIP3 may play a critical role in the protective effects of zinc during hyperglycemic conditions. However, the current observations are limited to in vitro experiments with excessive glucose concentrations. Other mechanisms contributing to the protective effects of zinc in this model cannot be ruled out. For example, zinc directly improves serum calcification propensity [33], which could also be reflected in calcification in vitro.

The anti-inflammatory and anti-calcific effects of zinc could be relevant to diabetes mellitus, a condition associated with impaired zinc homeostasis [26]. However, the effects of zinc on NF-kB may be cell-specific [48]. The beneficial effects of zinc supplementation on glycemic control are discussed in [62]. Interestingly, GPR39-deficient mice fed a high-fat/high-sucrose or low-fat/high-sucrose diet showed increased prandial glucose levels [63]. Beyond glycemic control, zinc supplementation may exert beneficial effects on cardiovascular inflammation, a key mechanism promoting vascular calcification [18,64]. In the diabetic db/db mouse, zinc deficiency exacerbates diabetic cardiomyopathy [65]. Similarly, cardiac inflammation in mice fed a high-fat diet is reduced by zinc supplementation [66]. In LDL receptor knockout mice, zinc supplementation exerts positive effects on the serum lipid profile [67]. A favorable effect of zinc supplementation on the lipid profile in humans was suggested in a previous meta-analysis [68]. Zinc supplementation was further found to prevent the full development of diabetic nephropathy in a mouse model [69]. A recent meta-analysis suggests that zinc supplementation may favorably influence risk factors for type 2 diabetes and cardiovascular disease [70]. In diabetic patients with coronary heart disease, the combined supplementation of magnesium and zinc has been associated with favorable effects [71]. Based on these and other beneficial effects, zinc supplementation has been widely discussed as a therapeutic tool in diabetes and other pathologies [72], a concept further supported by the current observations. However, the artificial environment of primary VSMCs in culture warrants caution when translating effects to diabetic patients. Further research in vivo is required to confirm the protective effects of zinc on diabetes-related vascular calcification.

In conclusion, zinc supplementation blunts the pro-calcific effects of excessive extracellular glucose concentrations in VSMCs, an effect associated with impaired high glucose-induced NF-kB activation. The effects of zinc in VSMCs appear to be dependent on GPR39 and TNFAIP3. Further studies addressing whether zinc supplementation may ultimately translate into better cardiovascular outcomes in diabetes mellitus are warranted.

## Data Availability

The data presented in this study are available from the corresponding author on reasonable request.
